# Combining target enrichment with barcode multiplexing for high throughput SNP discovery

**DOI:** 10.1186/1471-2164-11-641

**Published:** 2010-11-18

**Authors:** Nik Cummings, Rob King, Andre Rickers, Antony Kaspi, Sebastian Lunke, Izhak Haviv, Jeremy BM Jowett

**Affiliations:** 1 Genomics and Systems Biology, Baker IDI Heart and Diabetes Institute, Melbourne, Australia; 2 Systems Integrations, Baker IDI Heart and Diabetes Institute, Melbourne, Australia; 3 Human Epigenetics, Baker IDI Heart and Diabetes Institute, Melbourne, Australia; 4 DNA and Blood Profiling Facility, Baker IDI Heart and Diabetes Institute, Melbourne, Australia; 5 GeneWorks Pty Ltd, Adelaide, Australia; 6 Department of Biochemistry, School of Medicine, University of Melbourne, Melbourne, Australia; 7 Metastasis Research Laboratory, Peter MacCallum Cancer Centre, Melbourne, Australia

## Abstract

**Background:**

The primary goal of genetic linkage analysis is to identify genes affecting a phenotypic trait. After localisation of the linkage region, efficient genetic dissection of the disease linked loci requires that functional variants are identified across the loci. These functional variations are difficult to detect due to extent of genetic diversity and, to date, incomplete cataloguing of the large number of variants present both within and between populations. Massively parallel sequencing platforms offer unprecedented capacity for variant discovery, however the number of samples analysed are still limited by cost per sample. Some progress has been made in reducing the cost of resequencing using either multiplexing methodologies or through the utilisation of targeted enrichment technologies which provide the ability to resequence genomic areas of interest rather that full genome sequencing.

**Results:**

We developed a method that combines current multiplexing methodologies with a solution-based target enrichment method to further reduce the cost of resequencing where region-specific sequencing is required. Our multiplex/enrichment strategy produced high quality data with nominal reduction of sequencing depth. We undertook a genotyping study and were successful in the discovery of novel SNP alleles in all samples at uniplex, duplex and pentaplex levels.

**Conclusion:**

Our work describes the successful combination of a targeted enrichment method and index barcode multiplexing to reduce costs, time and labour associated with processing large sample sets. Furthermore, we have shown that the sequencing depth obtained is adequate for credible SNP genotyping analysis at uniplex, duplex and pentaplex levels.

## Background

The development of massively parallel sequencing or next generation sequencing (NGS) platforms provide the capacity for high-throughput sequencing of whole genomes at low cost. However, while those platforms improve the capacity to find novel variations that are not covered by existing genotyping arrays, they do not make use of the existing data, composed of thousands of relatively small genomic regions that have been associated with diseases through the use of genome wide association and linkage studies, where isolation of causative genetic variants has been problematic.

The efficiency of NGS-mediated genotyping has recently been improved through employing amplicon libraries of long-range PCR, which encompass discrete genomic intervals [1]. However, this method of library construction remains time-consuming, costly and limited to very small genomic regions (5 kbp-1 Mbp) and is impractical for genetic dissection of disease linked loci which can span 10 Mb or more. The development of molecular inversion probes (MIPs) and the use of chip-based technologies for massively parallel capture of specific genomic targets is limited by representational and allelic bias and remains costly and time consuming [2,3]. Recent advances in genome enrichment technologies provide efficient methods of region-specific, in-solution partitioning of regions spanning several megabases [4,5]. These technologies can be used to "capture" whole contiguous regions or generate exon specific libraries for discovery of functional variants within regions determined by genome wide association and linkage studies. However, the efficiency of genome partitioning and NGS is also compromised, since at the current size of capture for example up to 10 MB, the depth of read for a single sample, through a single lane of Illumina GAIIx (50-170 fold coverage), far exceeds that needed for confident SNP calling.

Nucleotide-based barcodes have been used to multiplex individual samples for use on NGS platforms [1,6-9]. This methodology exploits the sequencing depth of NGS technologies to sequence multiple samples in a single flow cell, reducing costs and increasing throughput. While these methodologies represent a significant advance in resequencing throughput, they do not provide the ability to target sequencing to specific disease-linked loci. The importance of continued sequencing efforts, particularly in focused populations, to analyse the differences between disease-affected and unaffected individuals has been recognised [10]. To achieve this outcome, the next logical step has been to combine multiplexing barcode technology with targeted enrichment. This has the effect of focussing the power of NGS sequencing onto a particular region (which contains putative disease genes), and simultaneously allowing pooling and later de-convolution of individual DNA samples. The outcome of this is to increase the throughput and significantly reduce the cost of resequencing disease-linked loci in large cohorts.

Our experimental data describes a cost effective, high throughput method for region-specific, multiplex sequencing by combining genome partitioning and barcode indexing with NGS technology. Individual samples were multiplexed prior to sequencing and successfully separated *in silico *after sequencing on the Illumina GAII platform. Genomic libraries were selectively enriched for the human chromosome X exome using Agilent's SureSelect methodology and resulting sequences were efficiently mapped to the human X chromosome.

## Results

**Figure 1:**
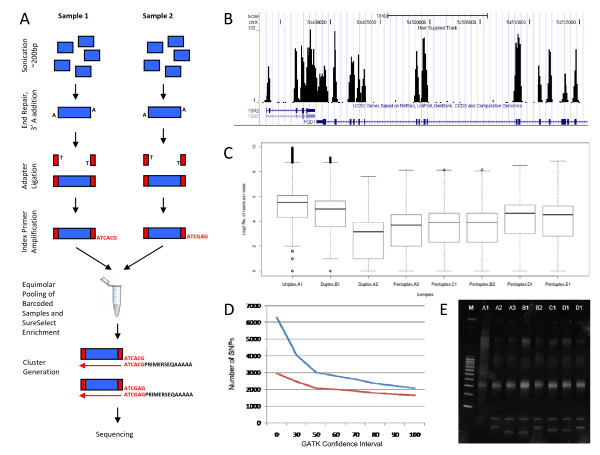
**Massively parallel sequencing of samples enriched for exons on chromosome X following addition of index primers to allow multiplexing**. **(A) **Workflow of procedure showing library construction, adaptor/index ligation, amplification and target enrichment. **(B) **Plot of number of reads per base across region of the chromosome X showing highly focussed sequence reads around exons and low background outside the target regions. **(C) **The box-and-whisker plot to examine the distribution of read coverage (log 2 of reads per base) for each of the 8 samples in uniplex, duplex and pentaplex sequencing lanes. The x-axis represents the individual lanes, while the y-axis represents the number of superimposed reads. Boxes represent the interquartile range, with the 75th percentile at the top and the 25th percentile at the bottom. The line in the middle of the box represents the 50th percentile, or median. Whiskers represent the rest of the distribution, with their terminations representing the lowest and highest feature intensity values. Box-and-whisker plots were performed for each sample in either level of plexity. **(D) **Effect of varying the confidence parameter in GATK analysis software on total number of SNPs called (blue line) and number of SNPs annotated in dbSNP database (red line). Representative example shown is sample D1 from the pentaplex. **(E) **Confirmation of DNA fragmentation and library construction by agarose gel electrophoresis confirming size range of each index amplified library.

Eight genomic DNA samples from 5 individuals (labelled A1, A2, A3, B1, B2, C1, D1 and E1) were used. All samples were processed in the same manner except sample A1, which was not indexed, but was partitioned and sequenced as a uniplex. Samples A2 and B1 were indexed, pooled, partitioned and sequenced as a duplex. Samples A3, B2, C1, D1 and E1 were indexed, pooled, partitioned and sequenced as a pentaplex. Samples A, B and E were taken from male participants and samples C and D were from female participants to allow for the influence of chromosome X copy number on partitioning results. This design allows us to determine (1) the efficiency of deconvoluting individual samples from a multiplex at duplex and pentaplex levels (2) sequence consistency between uniplex and multiplex samples and (3) the effect of multiplex indexing on target enrichment. A schematic representation of sample preparation is shown in Figure 1A. Barcode indexing was performed with indexing primers from Illumina (Illumina, California, USA) and GeneWorks (GeneWorks, Adelaide, Australia) and genome partitioning was performed using SureSelect Human Chromosome X Exome Kit (Agilent, California, USA).

To test the feasibility and utility of multiplexing individuals for genotyping through genomic partitioning and NGS, we compared the genotyping data of the same individual, in the context of uniplex, duplex, and pentaplex genomic mixes. We prepared genomic DNA from white blood cells of five individuals, fragmented the DNA, and constructed three adaptor ligated libraries, each for one Illumina GAII lane. In one lane, the individual was genotyped as uniplex, in the second, as duplex with another individual, and in the third lane, as pentaplex with four additional individuals. The single individual genomes (total of eight) were tracked through the inclusion of six mer indices that is unique to each individual.

Sequence generation and processing was performed on the Illumina GAII platform using Illumina pipeline (v 1.3.2). Sequencing was performed as 65 bp single-end reads. Sequences were aligned to the hg18 reference genome using the BWA aligner (v 0.5.5) [11]. Resulting files were modified with the Picard toolkit [12] before SNP analysis was undertaken using Genome Analysis Toolkit (GATK). A comprehensive description of library preparation, target enrichment and bioinformatics is provided in the Materials and Methods section of this paper.

Analysis of the uniplex data (sample A1) shows 16,403,360 unique sequences across the human genome. Of these, 26% of sequences were mapped to the X chromosome. The baits for target enrichment were designed for exons within the X chromosome totalling 3.3 Mb. 88% of sequence reads map on or within 500 bp of targeted exons. This represents 97-fold enrichment of the X chromosome exons (Table 1). An example of tiled sequence aligning to X chromosome exons is shown in Figure 1B. The genome browser clearly shows the vast over-representation of reads from the genome that span the target areas and low "background" reads outside of these areas. 2,399 SNPs were discovered on the X chromosome, 1,839 (77%) of these were found to be previously annotated on dbSNP and 560 were novel SNPs (Table 2). We used a GATK confidence parameter of 70, which minimises the discovery of the false positives, yet retains identification of novel SNPs. The parameter was chosen by comparing the converging plots of total number of SNPs detected and the proportion of these annotated on dbSNP (Figure 1D). With increasing stringency the two plots tend toward parallel with the difference comprising novel SNPs. All plots were similar, a representative plot is shown (sample D1 from pentaplex).

**Table 1:** Sequencing and target enrichment results for 8 samples in uniplex, duplex and pentaplex reactions.

Sample	Plexity	Filtered Reads	Mapped to ChrX	Mapped to Target Region	% Mapped to X	% Mapped to Target	Fold Enrichment
A1	Uniplex	16,403,360	4,044,511	3,564,834	25.6	22.5	97
A2	Duplex	2,035,467	730,156	666,572	36.9	33.7	170
B1	Duplex	11,833,641	2,655,666	2,313,476	23.2	20.2	85
A3	Pentaplex	3,037,781	1,131,596	1,036,600	38.1	34.9	180
B2	Pentaplex	3,204,352	1,230,630	1,129,857	39.3	36.0	190
C1	Pentaplex	3,122,661	1,220,017	1,123,547	40.1	36.9	196
D1	Pentaplex	3,687,146	1,944,667	1,778,223	53.8	49.2	324
E1	Pentaplex	3,078,897	1,832,550	1,696,950	60.6	56.1	427

**Table 2:** Variant identification results across target region by sample.

Sample	Plexity	Total SNPs*	Annotated	Novel	% Annotated
A1	Uniplex	2,399	1,839	560	77%
A2	Duplex	1,462	991	471	68%
B1	Duplex	2,385	1,627	758	68%
A3	Pentaplex	2,102	1,242	860	59%
B2	Pentaplex	1,910	1,289	621	67%
C1	Pentaplex	2,033	1,362	671	67%
D1	Pentaplex	2,600	1,904	696	73%
E1	Pentaplex	2,390	1,760	630	74%

* Calculations performed using confidence parameter of 70 in GATK.

Duplex data (samples A2 and B1) were split by index barcode and analysed independently. For sample A2, there were 2,035,467 unique sequences across the human genome. Of these, 37% of sequences were mapped to the X chromosome, of these 91% of sequence reads map on or within 500 bp of targeted exons. This represents 170-fold enrichment of the X chromosome exons (Table 1). 1,462 SNPs were discovered on the X chromosome. Of these, 991 (68%) were found to be previously annotated on dbSNP and 471 were novel SNPs (Table 2). For sample B1, there were 11,833,641 unique sequences across the human genome. Of these, 23% of sequences were mapped to the X chromosome, 87% of these mapped on or within 500 bp of targeted exons. This represents 85-fold enrichment of the X chromosome exons (see Table 1). 2,385 SNPs were discovered on the X chromosome. Of these, 1,627 (68%) were found to be previously annotated on dbSNP and 758 were novel SNPs (see Table 2). The discrepancy between the two individual samples within the duplex is attributable to quantification error. This technique is sensitive to quantification which can, in turn, affect the accuracy of equimolar pooling of multiplexed samples. We employed Nanodrop quantification to determine individual library concentrations however, PicoGreen-based quantification would be more effective at this critical point.

Pentaplex data (samples A3, B2, C1, D1 and E1) were split by index barcode and analysed independently. For individual samples, there was a median of 3,122,661 ± 264,985 unique sequences across the human genome. Of these, 40 ± 10% of sequences were mapped to the X chromosome, of these 91 ± 0.5% of sequence reads map on or within 500 bp of targeted exons. This represents an average 263-fold enrichment of the X chromosome exons for each of the 5 individual samples in the pentaplex (Table 1). 2,102 ± 281 SNPs were discovered on the X chromosome. Of these, 1,362 ± 300 were found to be previously annotated on dbSNP and 671 ± 96 were novel SNPs (Table 2). For sample A and B, which were genotyped three and two times, respectively, we found 2102 ± 478 and 2147 ± 335 SNPs, respectively.

We found that the overall depth of read across all targeted bases was comparable between the uniplex and pentaplex versions of individual A (Figure 1C). Although deeper read depth provides higher confidence in SNP calling, our read depth across all samples was ~16X, which is adequate for the GATK algorithm to confidently call variant genotypes in the sample at a confidence interval of 70.

## Discussion and Conclusion

Our work benchmarks the use of a new target enrichment technology combined with a barcode indexing method with the aim of reducing the time and costs associated with NGS platforms. We have demonstrated the ability to genotype five individuals across a 3.3 Mb targeted region with confidence that we would detect the majority of the SNPs within the targeted region. Furthermore, we have shown that the sequencing depth obtained is adequate for credible SNP genotyping analysis at uniplex, duplex and pentaplex levels.

Our experiments describe re-sequencing of discontiguous exons where we observed that coverage per base was reduced at the flanks and increased at the middle of the sequenced regions (Figure 1B). Sequencing of contiguous regions, or larger blocks of discontiguous regions, would increase the average read depth across all samples. Additionally, with the introduction of high capacity sequencing technologies such as Illumina Hi Seq™, the read depth per multiplexed sample will increase dramatically providing higher confidence in SNP calling and the potential to increase plexity when read depth exceeds that required for confident genotyping. The successful demonstration of this methodology may facilitate the efficient identification of key genetic variants in disease linked loci.

## Methods

### Ethics Statement

All participants in this project provided informed consent for sample collection and retention. The project was approved by The Alfred Research and Ethics Unit (Approval number 312/10).

### Genomic DNA preparation and Library Construction

Whole blood was taken from 5 individuals (3 males and 2 females) and genomic DNA was extracted using QIAamp DNA Blood Midi Kit (Qiagen, Hilden, Germany). Individual genomic DNA samples were quantified by Nanodrop (Agilent, California, USA) and equalised to 30 ng/uL and 100 uL (3 ug) was sonically sheared to 200 bp on the Covaris S2 system (Covaris, Massachusetts, USA) with the following parameters:

Duty Cycle:   10%

Intensity:   5

Cycles per Burst:   200

Time (seconds):   180

Nucleotide overhangs produced as a result of the shearing process were converted to blunt ends using Klenow enzyme. The end-repair mix contained 27 uL DNA sample, 48 uL water, 10 uLT4 DNA Ligase buffer with 10 mM ATP, 4 uL 10 mM dNTP mix, 5 uL T4 DNA polymerase, 1 uL Klenow enzyme and 5 uL T4 PNK. Samples were incubated in a thermocycler at 20°C for 30 minutes. After incubation, samples were purified using QIAquick columns from a QIAquick PCR Purification Kit (Qiagen, Hilden, Germany) and eluted in 32 uL of Qiagen buffer EB.

Fragments were prepared for adapter ligation by addition of dATP to the 3' end of the blunt phosphorylated DNA fragments. A 50 ul reaction mix was made for each sample containing 32 uL DNA sample, 5 uL Klenow buffer, 10 uL 1 mM dATP and 3 uL Klenow fragment (3' to 5' exo minus). The reactions were incubated at 37°C for 30 minutes. After incubation, samples were purified using QIAquick MinElute columns from a MinElute PCR Purification Kit (Qiagen, Hilden, Germany) and eluted in 10 uL of Qiagen buffer EB.

Index PE adapters (Multiplexing Sample Preparation Oligonucleotide Kit, PE-400-1001, Illumina, San Diego, USA) were ligated to the ends of the DNA fragments at a 10:1 molar ratio of adapter to insert DNA for all samples except A1. For sample A1, paired-end adapters (Paired End DNA Sample Preparation Kit, FC-102-1001, Illumina, San Diego, USA) was used at the same molar ratio as the other samples. A 50 ul reaction mix was made for each sample containing 10 uL DNA sample, 25 uL 2X DNA ligase buffer, 10 uL Index PE adapter oligo mix and 5 uL DNA ligase. Samples were incubated in a thermocycler at 20°C for 15 minutes. After incubation, samples were purified using QIAquick columns from a QIAquick PCR Purification Kit (Qiagen, Hilden, Germany) and eluted in 30 uL of Qiagen buffer EB.

Samples were individually purified on 2% HR agarose (Ambion, Texas, USA) gels in Tris-Acetate-EDTA buffer to remove unligated and self-ligated adapters and for size selection of products for cluster generation. A 100 bp DNA ladder (GeneWorks, Adelaide, Australia) was used for size selection. Agarose blocks corresponding to the 300-320 bp were extracted using an x-tracta device (LabGadget, Illinios, USA). Samples were purified using QIAquick Gel Extraction Kit (Qiagen, Hilden, Germany) and eluted in 30 uL Qiagen buffer EB.

PCR was performed to selectively enrich each sample for DNA fragments with adapters ligated to both ends. Index barcodes are also introduced in this PCR. Two primers are used to amplify samples and a third unique indexing primer containing the indexing barcode is used to for samples discrimination after multiplex sequencing. A 50 uL PCR reaction mix was produced containing 5 uL DNA, 25 uL Phusion DNA polymerase, 1 uL PCR primer InPE1.0, 1 uL PCR primer InPE2.0, 17 uL water and 1 uL PCR index primer. Individual sample mixes contained sample specific PCR index primer. PCR was performed on a PTC-200 thermocycler (MJ Research, Massachusetts, USA) with the following cycle: 30 seconds at 98°C, then 14 cycles of 10 seconds at 98°C, 30 seconds at 65°C, 30 seconds at 72°C, followed by a final extension step of 5 minutes at 72°C and a hold step at 4°C. Sample A1 was amplified using PE PCR Primers 1.0 and 2.0. Samples A2 - E1 were amplified using PCR Primer InPE 1.0 and 2.0 and PCR Index Primers 4 - 10 respectively. After PCR, samples were purified using QIAquick columns from a QIAquick PCR Purification Kit (Qiagen, Hilden, Germany) and eluted in 50 uL of Qiagen buffer EB.

The concentration of each library was determined by measuring absorbance and confirmed for downstream processing if the OD 260/280 was approximately 1.8. After concentrations were determined, 1 ul of each library was analysed on 4-20% acrylamide/TBE Novex gel (Invitrogen, California, USA) alongside a 100 bp DNA ladder (GeneWorks, Adelaide, Australia) and visualised with SYBR Gold to confirm the size range of the library (see Figure 1E).

### Target Enrichment

Genome partitioning was performed using SureSelect Target Enrichment Kit and SureSelect Human Chromosome X Exome Kit (Agilent, California, USA). Individual samples were pooled at equimolar ratios so the final pooled multiplex library concentration was 147 ng/uL. A hybridisation buffer master mix was prepared for 3 library captures at room temperature containing 75 uL SureSelect hybridisation buffer #1, 3 uL SureSelect hybridisation buffer #2, 30 uL SureSelect hybridisation buffer #3 and 39 uL SureSelect hybridisation buffer #4. 40 uL of master mix was aliquoted into row A of a 96-well PCR plate. 3.4 uL of each library was aliquoted into row B. To each well of row B, 2.5 uL SureSelect Block #1, 2.5 uL SureSelect Block #2 and 0.6 uL SureSelect Block #3 was added and mixed by pipetting. Wells were sealed with strip caps and samples were incubated on a thermocycler at 95°C for 5 minutes and 65°C for 5 minutes. An oligo capture library was prepared in PCR strip tubes by adding 5 uL SureSelect Oligo Capture Library, 1 uL nuclease-free water and 1 uL RNase block to each of 3 tubes. The capture library was added to row C of the PCR plate, sealed with strip caps and the plate was incubated at 65°C for 2 minutes. After incubation, 13 uL of hybridisation buffer mix was taken from row A and added to row C and 7 uL of prepared library from row B was added to row C. The contents of row C were mixed by pipetting and the plate was sealed with plate sealing film. The hybridisation mixture was incubated at 65°C for 48 hours.

Dynabead M-280 Streptavidin (Invitrogen, California, USA) was prepared by vortexing and adding 50 ul Dynabeads to a 1.5 mL microfuge tube for each capture library. Dynabeads were washed 3 times by adding 200 uL SureSelect binding buffer, mixing on a vortex for 5 seconds, applying tube contents to magnetic separation and removing supernatant. After 3 washes, beads were resuspended in 200 uL SureSelect binding buffer. Each hybridisation mixture was added to a Dynabead solution after hybridisation and each tube was mixed by inverting 5 times. Each hybrid capture/bead solution was incubated at room temperature on a nutator for 30 minutes. Beads and buffer were then separated by magnetic separation and the supernatant was removed. Beads were resuspended in 500 uL SureSelect wash buffer #1 by vortex mixing for 5 seconds and incubated for 15 minutes at room temperature. Beads were washed 3 times by applying tube contents to magnetic separation, removing supernatant, adding 500 uL SureSelect wash buffer #2 (prewarmed to 65°C), mixing on a vortex for 5 seconds, incubating samples at 65°C for 10 minutes and inverting tube to mix. After 3 washes, beads were resuspended in 50 uL SureSelect elution buffer and mixed by vortexing for 5 seconds. All samples were incubated for 10 minutes at room temperature. Beads and buffer were then separated by magnetic separation and the buffer was moved to a new 1.5 mL microfuge tube. 50 uL SureSelect neutralisation buffer was added to the buffer.

Capture solution desalting was performed using a QIAquick MinElute PCR purification column from a MinElute PCR Purification Kit (Qiagen, Hilden, Germany). MinElute columns were brought to room temperature. 500 uL PBI was added to the sample solution and mixed by pipetting. Columns were placed in a 2 mL collection tube and 600 uL sample was added to the column. Samples were spun at 13,000 rpm for 60 seconds and flow-through was discarded. 750 uL of buffer PE was added to the column, samples were spun at 13,000 rpm for 60 seconds and flow-through was discarded. Column was spun again at 13,000 rpm for 60 seconds and column was placed in a new 1.5 mL microfuge tube. 15 uL buffer EB was applied directly to the MinElute filter and allowed to incubate for 60 seconds. Samples were spun at 13,000 rpm for 60 seconds and collected eluate (captured library) was stored at -20°C.

A post-hybridisation PCR was performed to amplify the index adapter ligated, genome partitioned samples. For each capture solution, an amplification reaction was performed using Phusion High Fidelity DNA Polymerase (Finnzymes, Espoo, Finland). The reaction mix contained 10 uL 5X Phusion High Fidelity buffer, 1.5 uL 10 mM dNTP mix, 1 uL 10 uM P5 primer, 1 uL 10 uM P7 primer, 1 unit Phusion DNA polymerase, 1 uL of captured DNA and 35 uL nuclease-free water. A PCR was performed on a PTC-200 thermocycler (MJ Research, Massachusetts, USA) with the following cycle: 30 seconds at 98°C, then 18 cycles of 10 seconds at 98°C, 30 seconds at 57°C, 30 seconds at 72°C, followed by a final extension step of 5 minutes at 72°C and a hold step at 4°C. Libraries were purified using QIAquick columns from a QIAquick MinElute Kit (Qiagen, Hilden, Germany) and eluted in 15 uL of Qiagen buffer EB and Tween20 (Sigma-Aldrich, Missouri, USA) was added to a final concentration of 0.1%. 1 uL of each library was analysed on 4-20% acrylamide/TBE Novex gel (Invitrogen, California, USA) alongside a 100 bp DNA ladder (GeneWorks, Adelaide, Australia) and visualised with Ethidium Bromide to confirm the size range and amplification of each library. Final library concentration measured by PicoGreen-based assay (Quant-iT PicoGreen dsDNA Assay Kit, Invitrogen) vs lambda DNA standard (Table 3).

**Table 3:** Concentrations of final multiplexed libraries for sequencing.

Library Number	Concentration (ng/uL)	Calculated nM*
1	12.7	57
2	11.8	53
3	5.8	26

*Calculation based on 340 bp average size and MW of one base pair = 660 g/mol.

### Sequence Generation

Libraries were diluted to 10 nM concentrations before further dilution to 4.5 pM for cluster generation and sequencing-by-synthesis on the Illumina Genome Analyser II (running SCS2.3/IPAR). Each library was sequenced on a single lane of an 8-lane flow-cell. GAII Images were processed using the Illumina Pipeline (v 1.3.2). Images were processed through Firecrest, producing signal intensity files. These signal intensities were processed by Bustard into base calls and ultimately sequence files. The uniplex had quality issues in the last 7 base pairs which prevented alignment against the reference genome. These problematic bases were trimmed off using the the fastx toolkit [13]. This caused the length of the uniplex sequences to drop from 64 bp to 57 bp long. The multiplexed samples were required to be split into separate sequence files for each sample, and the bar code sequence needed to be removed. This was done using the fastx toolkit [13], with a maximum bar code mismatch (edit distance) score of 2.

### Alignment and SNP calling

The sequence files were aligned against the hg18 reference genome using the BWA (v 0.5.5) aligner, using default parameters [11]. The resulting SAM files were sorted and compressed into BAM files by using the MergeSamFiles tool which is part of the Picard toolkit [12]. An index file for each BAM file was generated using the SAMTools index function [12]. The GATK - Unified Genotyper tool was used to generate a list of SNPs for each sample at different confidence levels (Figure 1D). These lists of SNPs where matched against dbSNP (build 130) [14] via Galaxy's Genomic Intervals tool kit [15]. This gave a list of identified SNPs which were known to dbSNP.

### Enrichment Analysis

The enrichment level of the targeted region was calculated by using the SAMTools pileup tool [12] to generate a count of reads on each individual base pair, and only select values which fell in the regions defined by the SureSelect design. The generated values were analysed in R [16] to generate the box plot (Figure 1C) and associated statistics. The fold enrichment factor was calculated by dividing the average base coverage value within the targeted region by the average base coverage value outside of the targeted region (background genomic reads). Average base coverage was the average number of times a base was read within the designated interval.

### Adapter and Primer Sequences

PE Genomic Adapters (Illumina, California, USA)

5' P-GATCGGAAGAGCGGTTCAGCAGGAATGCCGAG

5' ACACTCTTTCCCTACACGACGCTCTTCCGATCT

PE PCR Primers (used to amplify non-indexed sample A1)

PE PCR Primer 1.0 (Illumina, California, USA)

5'AATGATACGGCGACCACCGAGATCTACACTCTTTCCCTACACGACGCTCTTCCGATCT

PE PCR Primer 2.0 (Illumina, California, USA)

5'CAAGCAGAAGACGGCATACGAGATCGGTCTCGGCATTCCTGCTGAACCGCTCTTCCGATCT

Multiplexing Adapters (Illumina, California, USA)

5' P-GATCGGAAGAGCACACGTCT

5' ACACTCTTTCCCTACACGACGCTCTTCCGATCT

Multiplexing PCR Primers (Illumina, California, USA)

Multiplexing PCR Primer 1.0

5'AATGATACGGCGACCACCGAGATCTACACTCTTTCCCTACACGACGCTCTTCCGATCT

Multiplexing PCR Primer 2.0

5' GTGACTGGAGTTCAGACGTGTGCTCTTCCGATCT

PCR Indexing Primers (Illumina, California, USA)

PCR Primer Index 4: 5'CAAGCAGAAGACGGCATACGAGATTGGTCAGTGACTGGAGTTC

PCR Primer Index 5: 5'CAAGCAGAAGACGGCATACGAGATCACTGTGTGACTGGAGTTC

PCR Primer Index 6: 5'CAAGCAGAAGACGGCATACGAGATATTGGCGTGACTGGAGTTC

PCR Primer Index 7: 5'CAAGCAGAAGACGGCATACGAGATGATCTGGTGACTGGAGTTC

PCR Primer Index 8: 5'CAAGCAGAAGACGGCATACGAGATTCAAGTGTGACTGGAGTTC

PCR Primer Index 9: 5'CAAGCAGAAGACGGCATACGAGATCTGATCGTGACTGGAGTTC

PCR Primer Index 10: 5'CAAGCAGAAGACGGCATACGAGATAAGCTAGTGACTGGAGTTC

Illumina oligonucleotide sequences ^© ^2006-2008 Illumina, Inc. All rights reserved.

Flanking Post-Capture PCR Primers (GeneWorks, South Australia, Australia)

P5 Primer 5'-AATGATACGGCGACCACCG

P7 Primer 5'-CAAGCAGAAGACGGCATACGA

## Competing interests

The authors declare that they have no competing interests.

## Authors' contributions

NC, IH and JJ conceived of and designed the study. NC, RK and AR undertook the molecular genetic analysis. NC, AK, SL, IH and JJ analysed the sequence data. NC and JJ wrote the manuscript. All authors read, provided comments on and approved the manuscript.

## Funding

This work was supported by BakerIDI Heart and Diabetes Institute.
